# Variable expression of subclinical phenotypes instead of reduced penetrance in families with mild triphalangeal thumb phenotypes

**DOI:** 10.1136/jmedgenet-2019-106685

**Published:** 2020-03-16

**Authors:** Jacob W P Potuijt, Jeannette Hoogeboom, Esther de Graaff, Christianne A van Nieuwenhoven, Robert Jan H Galjaard

**Affiliations:** 1 Plastic, Reconstructive and Hand Surgery, Erasmus MC, University Medical Center Rotterdam, Rotterdam, The Netherlands; 2 Clinical Genetics, Erasmus MC, University Medical Center, Rotterdam, The Netherlands; 3 Division of Cell Biology, Neurobiology and Biophysics, Utrecht University, Utrecht, Utrecht, The Netherlands; 4 Plastic, Reconstructive and Hand Surgery, Erasmus MC, University Medical Centre Rotterdam, Rotterdam, The Netherlands

**Keywords:** polydactyly, genetic enhancer elements, congenital abnormalities

## Abstract

**Background:**

The of zone of polarizing activity regulatory sequence (ZRS) is a regulatory element residing in intron 5 of LMBR1 and regulates Sonic Hedgehog expression in the limb bud. Variants in the ZRS are generally fully penetrant and can cause triphalangeal thumb (TPT) and polydactyly in affected families.

**Objective:**

In this report, we describe two families with mild phenotypical presentation.

**Methods:**

We performed a field study for clinical evaluation and sequenced the ZRS for variantsusing Sanger sequencing.

**Results:**

In family I, a novel 165A>G variant in the ZRS (g.156584405A>G, GRCh37/Hg19) was found. In family II, we identified a 295T>C variant in the ZRS (g.156584535T>C, GRCh37/Hg19). Family members of both families who were presumed to be unaffected shared the variant in the ZRS with affected family members, suggesting reduced penetrance of the genotype. However, clinical examination of these unaffected family members revealed minor anomalies like broad thumbs and lack of thumb opposition. As the phenotype in affected patients is remarkably mild, we suggest that these ZRS variants are minimally disruptive for Sonic Hedgehog expression and therefore can result in subclinical phenotypes.

**Conclusion:**

Our study underlines the importance of accurate clinical examination and appropriate genetic counselling in families with mild cases of TPT.

## Introduction

Triphalangeal thumb (TPT) is a rare congenital hand anomaly in which the thumb has three phalanges instead of two. TPT is usually inherited in an autosomal dominant trait and is therefore commonly seen in affected families. In 1994, Heutink *et al* located the pathogenic locus of TPT at chromosome 7q36.^1^ Subsequently, Lettice *et al* determined that point mutations in the zone of polarising activity regulatory sequence (ZRS) causes TPT and preaxial polydactyly.^2^ The ZRS is a long-range regulatory element residing in intron 5 of *LMBR1* and regulates Sonic Hedgehog (*SHH*) expression in the embryonic limb bud. Since the identification of the ZRS region, 18 different point mutations in the ZRS have been reported in TPT families.^3^

There is broad phenotypical variability among different point mutations in the ZRS. For example, variants on locations 323 and 739 in the ZRS cause mild presentations of isolated TPT.^2 4^ Alternatively, severe anomalies such as TPT accompanied with tibial hypoplasia have been observed in families with variants on position 404 and 406 in the ZRS.^2 5–9^ In mildly affected phenotypes, reduced penetrance is regularly observed. In families who are more severely affected however, no reports of reduced penetrance have been made.

Identifying and reporting new variants in the ZRS is important for genotype-phenotype correlations in TPT families. Additionally, it will also help to further elucidate the exact molecular mechanism of the role of the ZRS in the regulation of *SHH* expression in the embryonic limb.

We therefore report two families with variants in the ZRS. These variants were identified in Dutch families with isolated TPT. Additionally, unaffected family members shared these variants with affected family members. Although this observation suggests that the genotype is not fully penetrant, minor anomalies within these presumed unaffected family members indicate subclinical expression of a TPT phenotype rather than reduced penetrance of the genotype. We define subclinical phenotypes as anomalies that are not recognised by affected family members since they do not cause functional constraints in daily life, but can be recognised during clinical workup by experienced physicians.

## Methods

### Clinical evaluation

**Figure 1:**
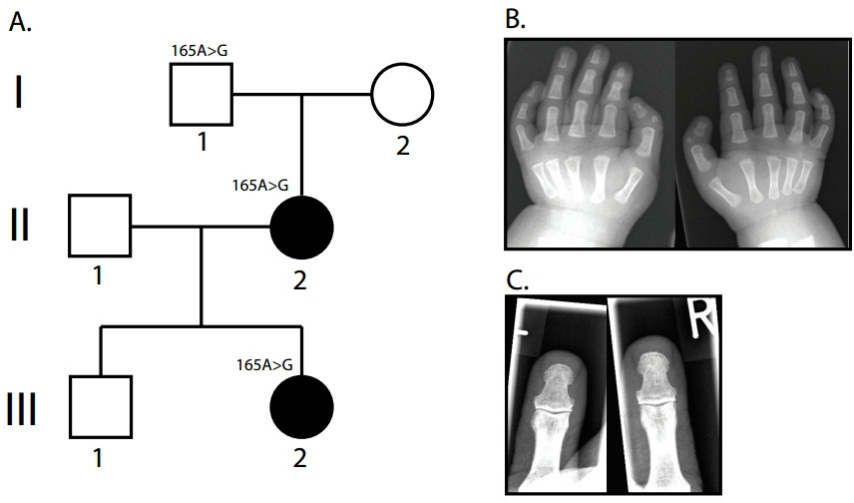
Overview of Dutch TPT family 1. (A) Pedigree of the Dutch TPT family 1. The index patient is patient III-2. (B) X-ray image of the hand of the index patient. An additional deltaphalanx is present in both thumbs. (C) X-ray image of the thumbs of patient III-2. Although there is no triphalangism present, the thumbs are remarkably broad. TPT, triphalangeal thumb.

Families 1 and 2 were identified at the outpatient clinic for Congenital Hand and Upper Limb Anomalies at the Sophia Children’s Hospital in Rotterdam, The Netherlands. The family members were clinically examined and consulted by a clinical geneticist. In family 1, peripheral blood samples were collected from the index patient, the mother and the grandfather of the index patient (figure 1). No blood samples were obtained from the brother of this patient as he was clinically unaffected and was below adult age.

**Figure 2:**
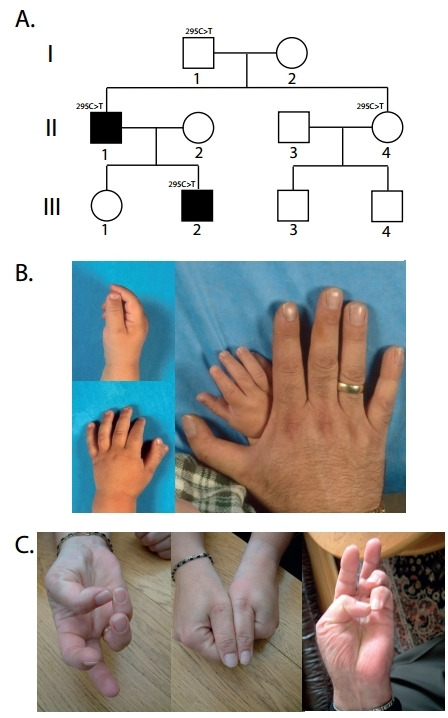
Overview of Dutch TPT family 2. (A) Outtake of pedigree of the Dutch TPT family 2. (B) Images of patient III-2 and his father (II-2), showing triphalangism of both thumbs with one additional ray on the left hand. (C) Images of patients II-4 and I-1, showing no triphalangism but lack of thumb opposition and mild thenar hypoplasia. TPT, triphalangeal thumb.

In family 2, the index patient (III-2) visited the outpatient clinic for Congenital Hand and Upper Limb Anomalies at the Sophia Children’s Hospital in Rotterdam with his parents. The other family members were visited as part of a field study. Included family members were clinically evaluated by a clinical geneticist, photographs were obtained and peripheral blood samples were collected (Figure 2, online supplementary figure 1). No radiographs were obtained during the field study.

10.1136/jmedgenet-2019-106685.supp1 Supplementary data

### ZRS sequencing

DNA samples were isolated from peripheral blood. The fragments were amplified using standard PCR. An 834 bp fragment covering the ZRS (774 bp) was sequenced in family members of both families (UCSC Genome Browser, hg19, chr7:156583766–156584600). Sequencing of PCR products was executed using Big Dye Terminator 3.1. Fragments were loaded on an ABI 3130 Sequence analyser and genetic analysis was performed with SeqScape Software (V.3.0).

## Results

### Clinical report

#### ​Family 1

Family 1 (figure 1A) consists of a nuclear family containing two affected patients with TPT. The index patient had a bilateral isolated TPT with an additional deltaphalanx (figure 1B). No other congenital hand or other anomalies were present. The mother of the index patient was born with a TPT accompanied with a rudimentary additional thumb on both hands, without any other hand or congenital anomaly (data not shown). The maternal grandfather of the index patient did not have a TPT or preaxial polydactyly. However, clinical examination of the hands revealed remarkable broadness of both thumbs and mild thenar hypoplasia. Although the X-ray image of the grandfather shows no duplication of the thumb or triphalangism, the broadness of the distal phalanges is striking (figure 1C).

#### ​Family 2

Family 2 comprises a large seven-generation family (Figure 2A, online supplementary figure 1). The index patient (III-2) had bilateral TPT with preaxial polydactyly on the left hand. The father of the index patient (II-1) had bilateral TPT without preaxial polydactyly (figure 2B). All other family members reported they were not affected. Although the thumbs of family members I-1 and II-2 did not show clear features of triphalangism, further examination revealed that both family members had mild thenar hypoplasia and were unable to oppose both thumbs (figure 2C). No other congenital anomalies were present in family 2.

### Mutation analysis

Sequence analysis of the 774 bp ZRS, in intron 5 of LMBR1, revealed the presence of a heterozygous A to G transition in members of family 1 (g.156584405A>G, GRCh37/Hg19). Following the more commonly used nomenclature for loci of ZRS variants, introduced by Lettice *et al*,^2^ this variant can be defined as a 165A>G variant.^2^ This variant was present in the affected family members. Patient I-1 of family one also carried a 165A>G variant in the ZRS, despite not having TPT on either hand. This variant was not present in public databases dbSNP, Clinvar and HGMD. Additionally, this variant was not present in locally available WGS data sets (GoNL, Wellderly, Public54).^10–12^

In family 2, we identified a 295T>variant in the ZRS (g.156584535T>C, GRCh37/Hg19). Two family members who did not have TPT carried the 295T>C variant. This variant has previously been reported in a British family with mild cases of TPT and reduced penetrance of the genotype.^13^ Additionally, transgenic enhancer assays in mice showed that the 295T>C variant causes ectopic expression in the embryonic limb and therefore confirms the pathogenicity of this variant.

## Discussion

In this brief report, we describe two TPT families with either a 165A>G or 295T>C variant in the ZRS. The aim of this paper was to show that these observations of reduced penetrance in TPT families are in retrospect caused by mild and subclinical limb phenotypes without the presence of triphalangism and therefore raise awareness for thorough clinical examination in members of TPT families who are presumed to be unaffected.

Ever since the identification of ZRS by Lettice *et al* in 2003, 18 variants in ZRS have been published in the literature.^2 4 6–9 13–20^ These variants are generally fully penetrant and have been found in families with either TPT or TPT with preaxial polydactyly. Exceptions to the above are point mutations on positions 105, 404 and 406 in ZRS, which cause more severe phenotypes like tibial hypoplasia and polysyndactyly.^2 5–9 21^

Although most variants in ZRS are considered fully penetrant, reduced penetrance has been reported in several TPT families with variants on positions 295, 334, 463 and 739 in ZRS.^13 14 16 17^

The first aim of this paper is to hypothesise that some of these observations might not be caused by reduced penetrance of the genotype, but by a subclinical expression of the phenotype. We base our hypothesis on two arguments. First, family members who were initially presumed unaffected do show minor anomalies or altered hand function when examined appropriately. In family 1 of this study, the grandfather did not have TPT but had evident broadness of the thumb. In family 2, patients with initially normal thumbs lacked the ability of opposition, which is caused by abnormal developmental patterning of the thumb. Although this observation is based on three patients from two families, we believe that these examples clearly illustrate our postulated hypothesis.

Second, reports of non-penetrance are consistently associated with mild phenotypes in TPT families and not with severe TPT phenotypes, like tibial hypoplasia and polysyndactyly. This indicates that these observations only occur in TPT families where *SHH* expression is only slightly disrupted. In these families, the variability in the phenotypical spectrum is apparently broad enough that family members with variants in ZRS can present with subclinical phenotypes instead of TPT. However, it remains unclear why the disruption of *SHH* causes TPT in one family member and a subclinical phenotype in another. One example of how intrafamilial variability can be explained is based on a reported family, where different degrees of somatic mosaicism were associated with various phenotypes in affected family members.^22^ As the regulatory function of ZRS on *SHH* is extremely delicate and affected by timing, location and level of activity, it is plausible that the slightest alteration of one of these factors can cause this interindividual phenotypical variation.

The second aim of this paper is to underline the importance of two aspects when clinically examining and counselling patients with an inherited type of TPT. First, it is important to clinically investigate the presumed unaffected family members, as these patients might not encounter functional problems in their daily life and will report they are unaffected. However, a distinct broadness of the thumb, a double flexion fold in the thumb or a duplicated lunula might indicate a discrete inclination for duplication of the thumb or the presence of an additional phalanx. Additionally, functional limitations regarding thumb strength or lack of opposition should be evaluated as well. Second, presumed unaffected family members should only be informed that their future offspring have a population-wide probability of having TPT or polydactyly after genetic evaluation. For complete reassurance, genetic evaluation of ZRS is also indicated for unaffected family members of mildly affected patients to verify whether they share the same disease-causing variant with their affected family members.
