# Supplementary figures and images for: Variable expression of subclinical phenotypes instead of reduced penetrance in families with mild triphalangeal thumb phenotypes
Source: J Med Genet. 2020 Mar 16;57(10):660–3. doi: 10.1136/jmedgenet-2019-106685 (PMC7525795; doi:10.1136/jmedgenet-2019-106685)

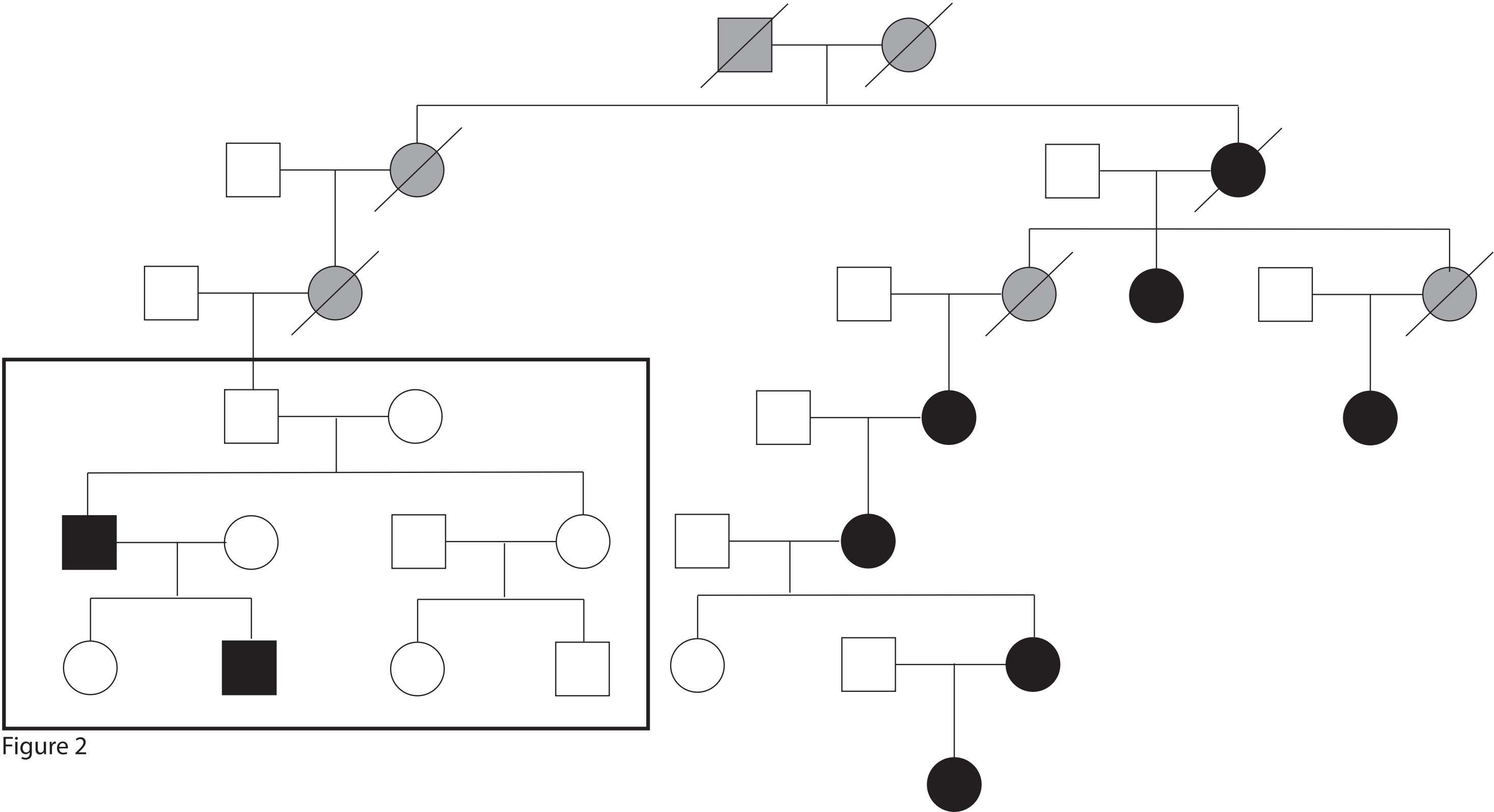

Figure 2

Supplement: Supplementary data [file jmedgenet-2019-106685supp001.pdf]
